# Supplementary figures and images for: Influence of Phosphodiesterase Inhibition on CRE- and EGR1-Dependent Transcription in a Mouse Hippocampal Cell Line
Source: Int J Mol Sci. 2020 Nov 17;21(22):8658. doi: 10.3390/ijms21228658 (PMC7696530; doi:10.3390/ijms21228658)

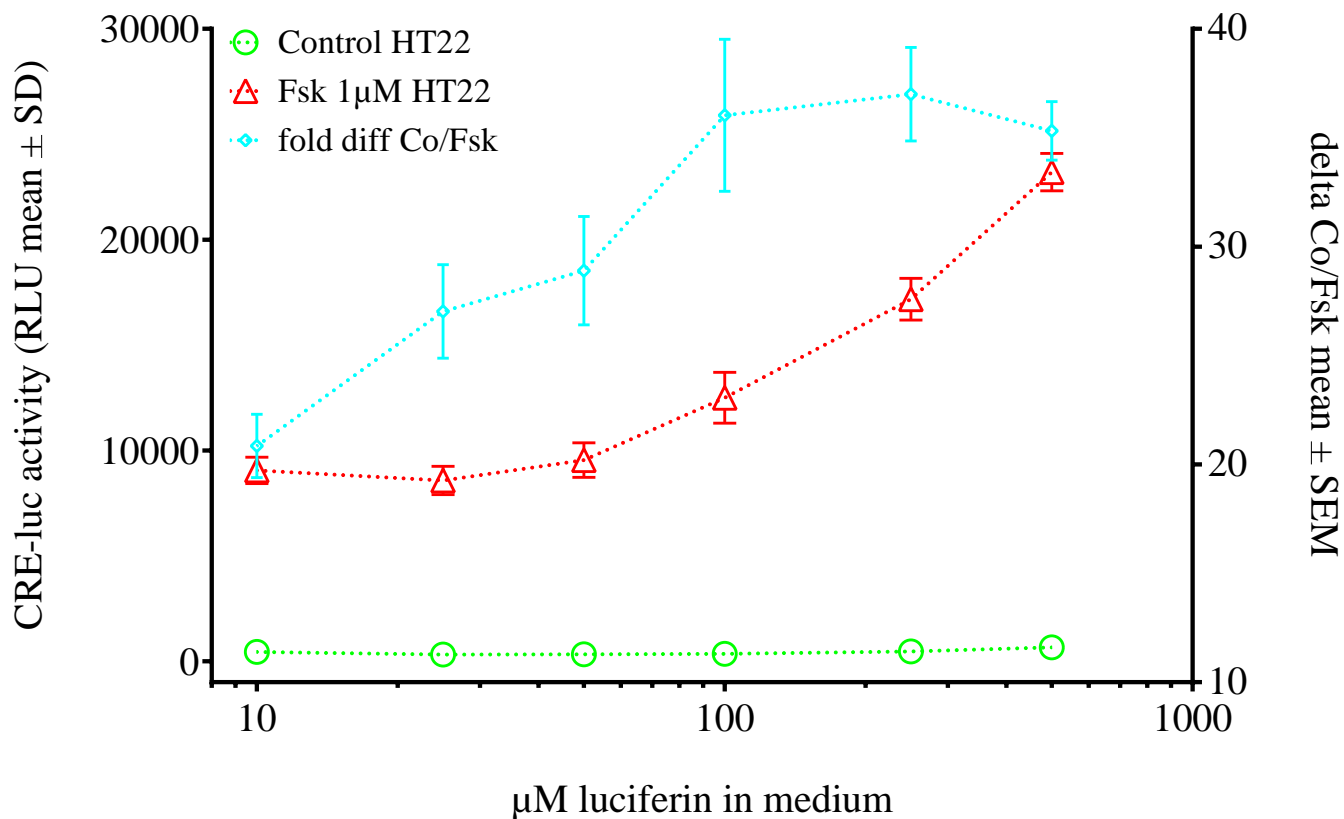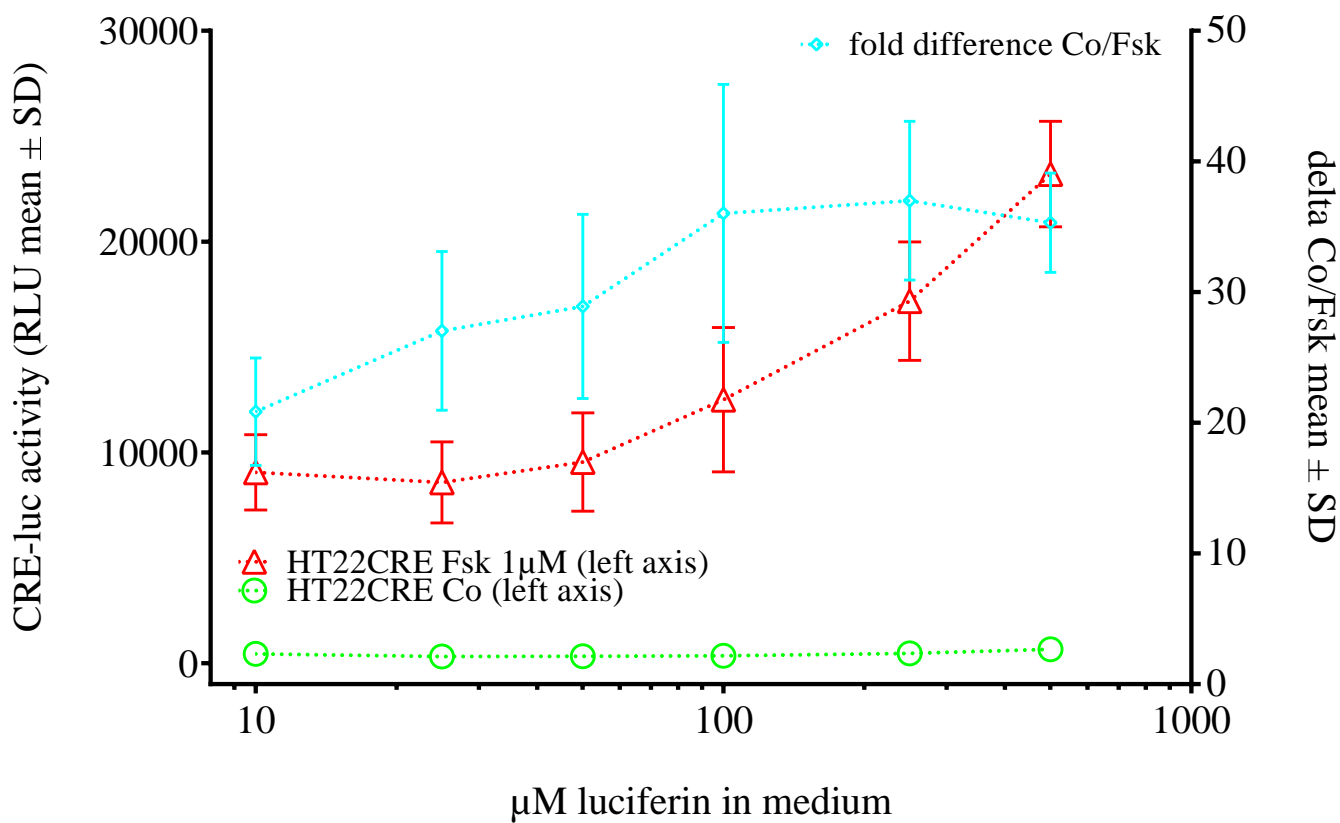

Supplement: Supplementary file 1 [file ijms-21-08658-s001.pdf]
